# Pneumothorax in Intubated Patients With COVID-19: A Case Series

**DOI:** 10.7759/cureus.31270

**Published:** 2022-11-08

**Authors:** Sanya Chandna, John Paul Colombo, Ankit Agrawal, Kavin Raj, Divyansh Bajaj, Sidharth Bhasin, Umesh Bhagat, Pooja Gogia, Balaji Yegneswaran

**Affiliations:** 1 Internal Medicine, Cleveland Clinic, Cleveland, USA; 2 Medical Research, St. Peter's University Hospital, New Brunswick, USA; 3 Medical Research, St. George's University School of Medicine, True Blue, GRD; 4 Cardiology, University of California, Riverside, USA; 5 Pulmonary and Critical Care, Medical College of Wisconsin, Milwaukee, USA; 6 Pulmonology, St. Peter’s University Hospital, New Brunswick, USA; 7 Hematology and Oncology, Maimonides Medical Center, New York City, USA; 8 Critical Care Medicine, St. Peter’s University Hospital, New Brunswick, USA

**Keywords:** pulmonary barotrauma, icu mortality, intubated, chest tube, pneumothorax (ptx), covid-19

## Abstract

Pneumothorax is a rare complication among mechanically ventilated patients since low tidal volumes are used nowadays instead of traditional high tidal volumes, but the incidence is slightly higher in patients with high positive end-expiratory pressure (PEEP). Herein we describe a case series of nine patients who were on mechanical ventilation due to acute respiratory distress syndrome (ARDS) secondary to coronavirus disease 2019 (COVID-19) and developed pneumothorax in due course. A retrospective analysis was done on COVID-19 intubated patients from March 2020 to June 2020 in a community hospital in Central New Jersey, which was one of the early hit states in the United States at the beginning of the pandemic. Outcomes were studied. The demographics of patients like age, gender, and body mass index (BMI); risk factors like smoking, comorbidities especially chronic lung disease, and the treatment they received were compared. We compared the total number of days on the ventilator, the highest PEEP they received, and the ventilator day when pneumothorax developed. All the patients who developed pneumothorax had a chest tube inserted to treat it. The mortality was noted to be 100% indicating that pneumothorax is a life-threatening complication of COVID-19 and COVID-19 by itself is a risk factor for pneumothorax likely due to a change in lung mechanics. There is a need for large-scale studies to confirm that these outcomes are related to COVID-19.

## Introduction

Pneumothorax is a rare complication among mechanically ventilated patients and is even higher in patients with high positive end-expiratory pressure (PEEP). While a primary spontaneous pneumothorax can occur without a precipitating event, a secondary spontaneous pneumothorax is a complication of underlying lung disease. Pneumothorax incidence increased in coronavirus disease 2019 (COVID-19) patients and was noted to be high, particularly in patients who were mechanically ventilated. Non-invasive ventilation modalities have a low barotrauma risk and associated complication rate. Now it is well known that radiographically COVID-19 pneumonia is characterized by ground glass opacities that progress to consolidative and fibrotic changes. Similarly, alterations like severe lung damage and diffuse alveolar damage explain how spontaneous pneumothorax complicates severe acute respiratory syndrome. Positive pressure ventilation's end-expiratory pressure raises the pressure gradient between the alveoli and the interstitial space and has the potential to rupture the alveoli, allowing air to extend into the pleura. In addition to barotrauma, COVID-19 reduces lung compliance which predisposes patients to develop pneumothorax as the condition progresses. Herein we describe a case series of nine patients intubated due to acute respiratory disease syndrome (ARDS) secondary to COVID-19 and developed pneumothorax in due course.

## Materials and methods

The charts of 71 COVID-19 patients who were admitted to the ICU in a community hospital in Central New Jersey, intubated, and placed on a mechanical ventilator (from March 1, 2020, to June 30, 2020) were reviewed retrospectively. All these patients presented with active COVID-19 illness symptoms and nasopharyngeal swabs were used to confirm the diagnosis of positive SARS-CoV-2 reverse transcription-polymerase chain reaction (RT-PCR). We included all the patients who experienced a pneumothorax while on a ventilator at any time. All the data was extracted manually from electronic medical records. Results were investigated. Comparisons were made between patient variables, such as ethnicity, age, gender, BMI, risk factors including smoking, and other comorbidities, particularly chronic lung disease. The total number of days spent on the ventilator, the highest PEEP they received, and the day a pneumothorax developed while on the ventilator were compared. Outcomes in COVID-19 intubated patients who experienced a pneumothorax were assessed.

## Results

Case 1

A 54-year-old obese Hispanic male with a medical history of hypertension and diabetes mellitus was brought to the emergency department (ED) for altered mental status (Table 1). On arrival, he was hypoxic to 19% on room air and rapid sequence intubation (RSI) was performed. He was placed on the ventilator with volume control settings. Inflammatory markers were elevated with a D-dimer of 381 ng/mL, C-reactive protein (CRP) 202 mg/L, lactate dehydrogenase (LDH) 534 U/L, and ferritin 248.3 ng/mL (Table 2). COVID-19 polymerase chain reaction (PCR) was positive. Chest x-ray (CXR) showed extensive diffuse bilateral alveolar infiltrates. The patient was consequently transferred to the intensive care unit (ICU) for further management.

**Table 1:** Baseline characteristics of the patients. HTN: hypertension, DM: diabetes mellitus, HLD: hyperlipidemia, ESRD: end-stage renal disease, PVD: peripheral vascular disease, RCC: renal cell carcinoma, COPD: chronic obstructive pulmonary disease, PEEP: positive end-expiratory pressure

Patient	Age (in years)	Sex	BMI (kg/m^2^)	Smoker	Chronic lung disease	Medical history	Total ventilator days	Highest PEEP before pneumothorax	Ventilator day when pneumothorax developed
1	54	Male	32	No	No	HTN, DM	44	14	14
2	55	Male	21.2	No	No	HTN, DM	58	12	21
3	41	Male	26.8	No	No	None	22	15	18
4	48	Male	30.2	No	No	HTN, DM, HLD, ESRD, PVD	7	10	2
5	48	Male	34.8	No	No	HLD	19	16	8
6	70	Male	NA	No	No	HTN, DM, HLD, RCC s/p nephrectomy	5	16	2
7	70	Female	31.9	Former	COPD	HTN, DM	9	14	5
8	66	Male	30.3	No	No	HTN	4	15	1
9	58	Male	34.1	No	No	None	42	16	7

**Table 2:** Laboratory investigations on admission.

	Total leukocyte count (10^3^×mm^3^)	Absolute lymphocyte count (10^3^×mm^3^)	Lactic acid (mmol/L)	D- dimer (ng/mL)	C-reactive protein (mg/L)	Lactate dehydrogenase (U/L)	Ferritin (ng/mL)
Reference values	4.0-11.0	1.0-3.0	0.5-2.0	0.00-211.00	0.0-5.0	140-271	18.0-464.0
Case 1	10.3	1.24	1.9	381	202	534	248.3
Case 2	17.5	1.40	6.8	3680	257	3434	NA
Case 3	13.8	1.10	NA	215	116	597	1460
Case 4	5.4	0.32	1.1	1484	300	756	7500
Case 5	13.3	1.06	4.3	6534	282	866	455
Case 6	7.2	0.72	3.0	299	198	459	NA
Case 7	6.4	0.38	NA	813	254	534	NA
Case 8	11.7	1.01	3.6	516	72	521	NA
Case 9	9.1	0.55	2.8	NA	288	612	NA

**Figure 1:**
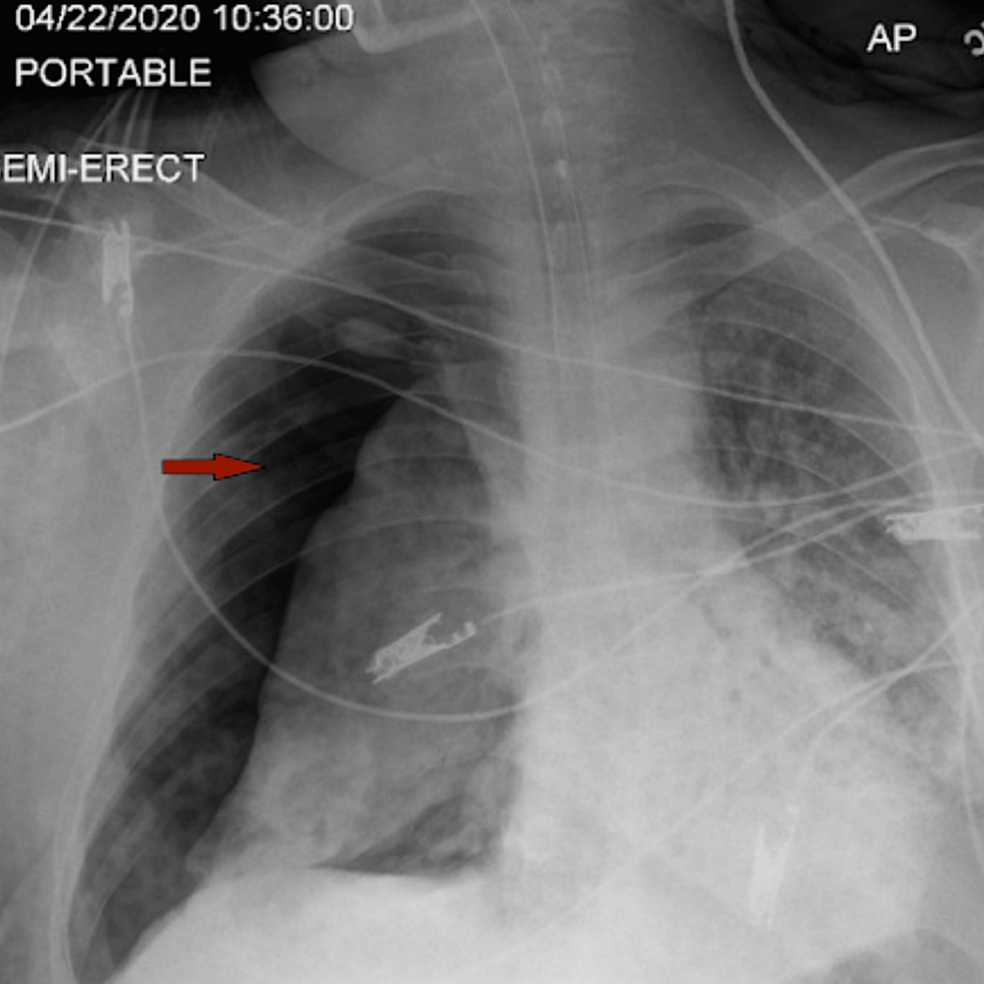
​​New large right-sided pneumothorax (arrow) with the collapse of the right lung.

**Figure 2:**
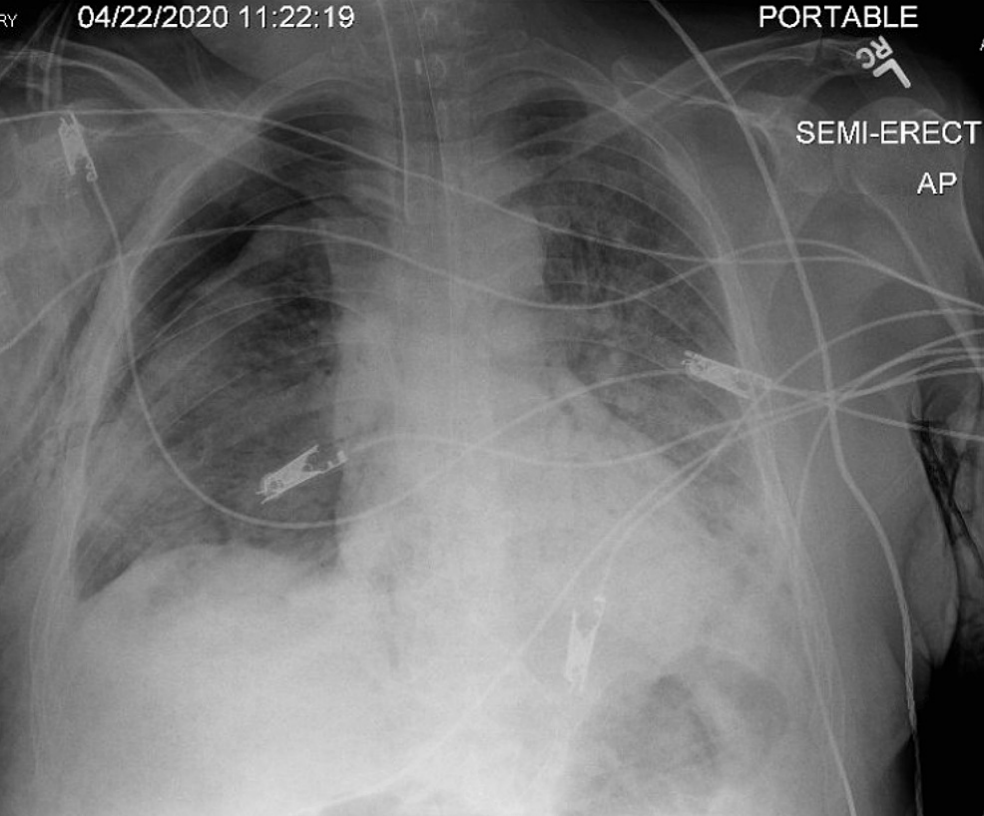
Large right pneumothorax with an improvement of collapsed right lung and tension component status post insertion of right chest tube.

**Figure 3:**
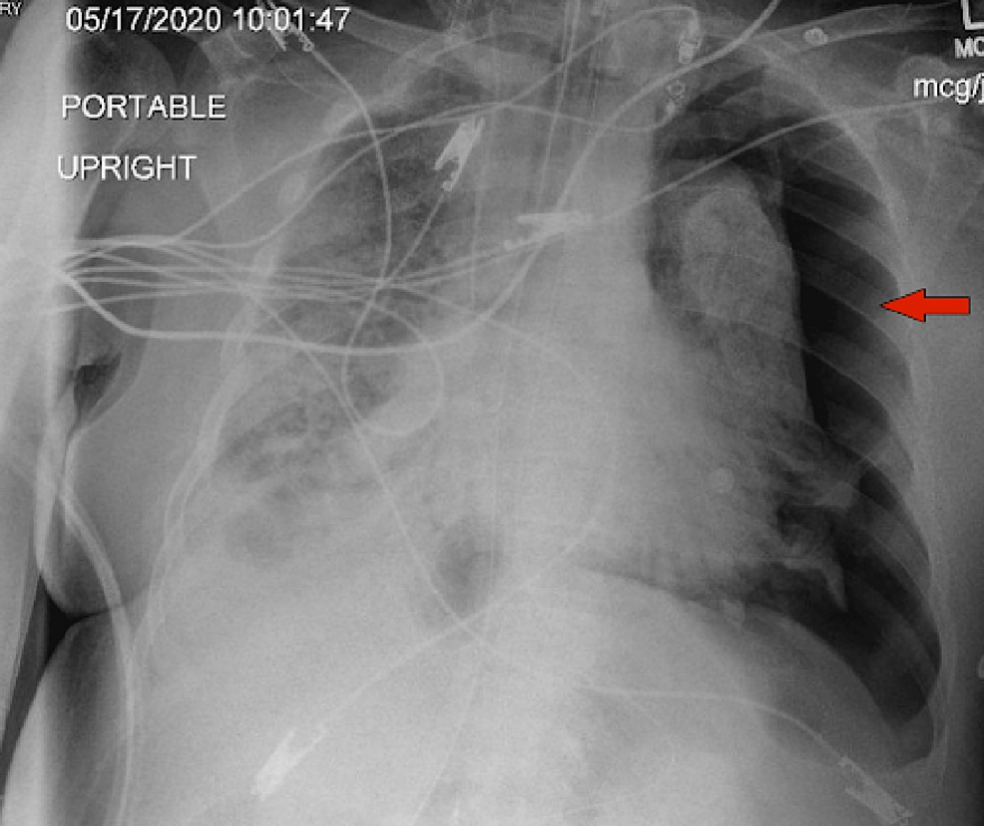
New large left pneumothorax (arrow) with a complete collapse of the left lung.

**Figure 4:**
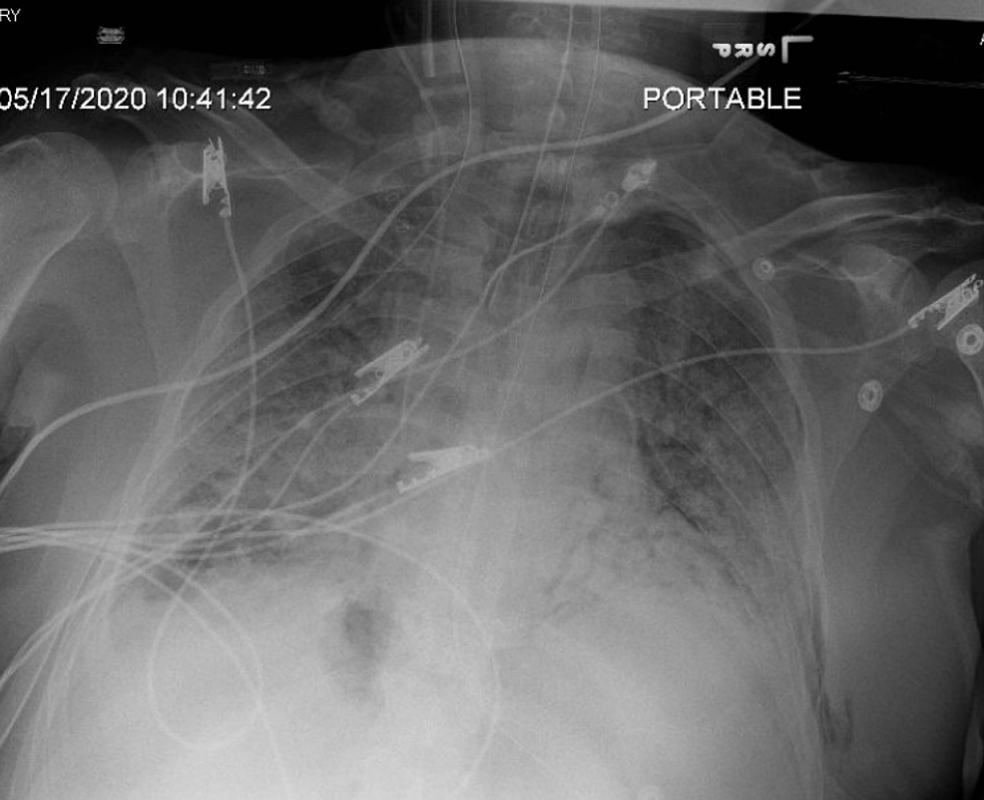
Interval placement of left chest tube. ​​Left pneumothorax is largely resolved with trace left apical gas noted within the left pleural space.

On the 14th day of the mechanical ventilation, he started getting hypoxic on the ventilator. Due to worsening oxygen saturation on the ventilator, a repeat CXR was obtained, revealing a new large right-side pneumothorax with the collapse of the right lung concerning tension pneumothorax (Figure 1). Ventilation settings around that time were volume control mode, respiratory rate (RR) of 30 breaths/min, PEEP of 12 cm H_2_O, tidal volume (TV) 430 mL, the fraction of inspired oxygen (FiO_2_) 100%, and inspiratory time (IT) 1 s. The highest PEEP on the ventilator since intubation was 14 cm H_2_O. Tube thoracostomy was performed with an 18 French (Fr) surgical catheter (Figure 2). Subsequently, he developed left pneumothorax with a complete left lung collapse (Figure 3) requiring a 16 Fr chest tube (Figure 4). Ventilator settings at the time of the second pneumothorax were volume control mode, RR of 30 breaths/min, PEEP 10 cm H_2_O, TV 340 mL, FiO_2_ 100%, and IT 1 s.

He had a protracted ICU stay which was complicated by superimposed methicillin-resistant *Staphylococcus aureus* pneumonia and bacteremia. He was treated with pressors, steroids, hydroxychloroquine, tocilizumab, broad-spectrum antibiotics, convalescent plasma, and full dose enoxaparin due to acute lower extremity deep venous thrombosis (DVT) in right soleal vein. On the 44th day of hospitalization, he had a cardiac arrest and eventually passed away.

Case 2

A 55-year-old Hispanic male with a medical history of hypertension and diabetes mellitus presented with shortness of breath of one-week duration (Table 1). His oxygen saturation was 70% on room air leading to RSI and mechanical ventilation with volume control settings. He was then transferred to the ICU. Biochemical investigations were significant for leukocytosis of 17.5×10^3^/mm^3^, D-dimer of 3680 ng/mL, CRP 257 mg/L, and LDH 3434 U/L (Table 2). CXR revealed diffuse patchy airspace opacities.

**Figure 5:**
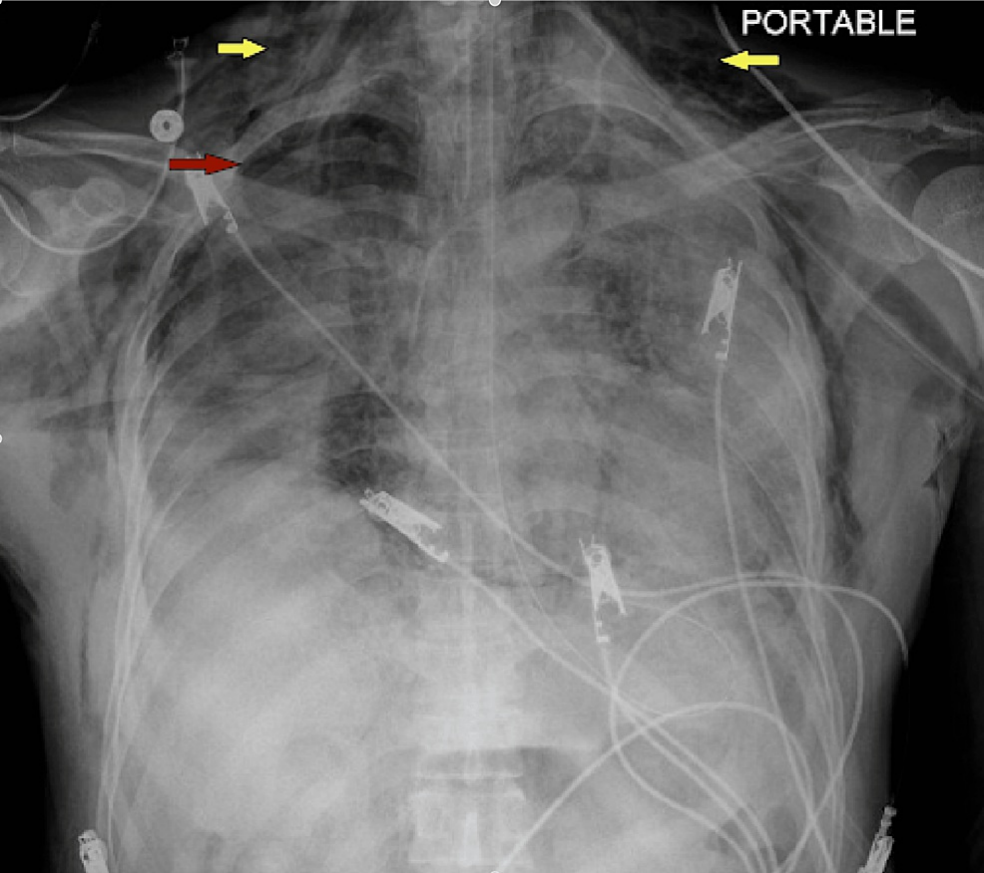
Small right-sided pneumothorax (red arrow), pneumomediastinum, bilateral subcutaneous emphysema (yellow arrow) involving the neck and chest wall.

**Figure 6:**
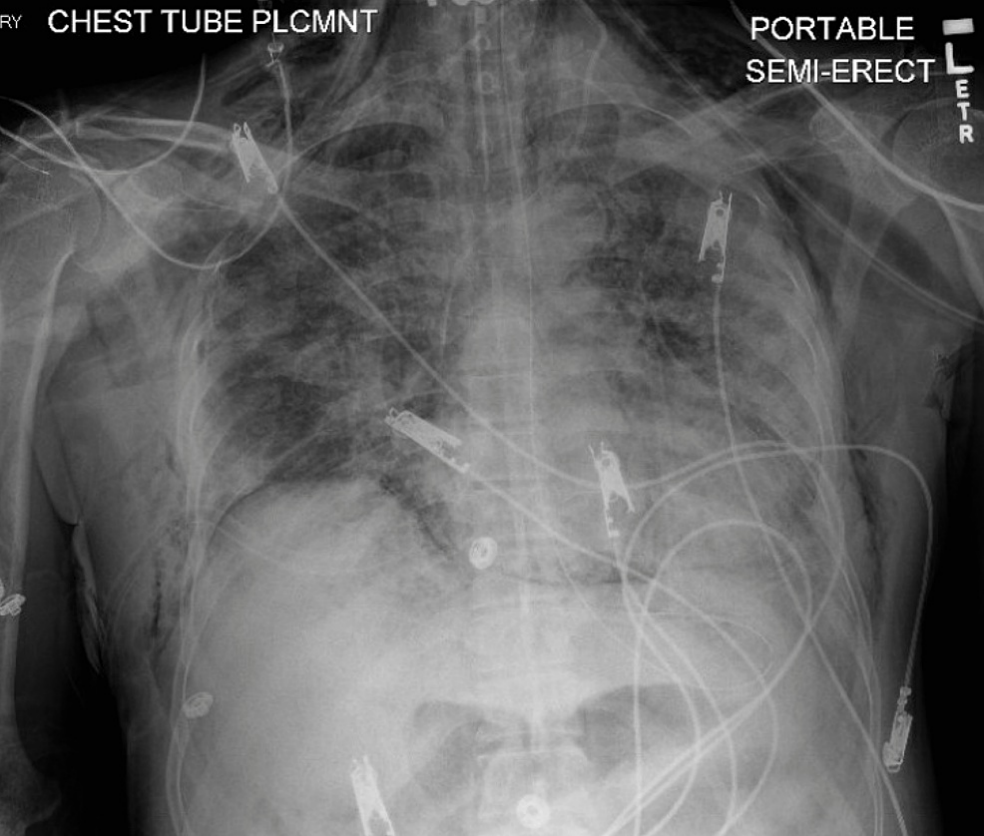
New right chest tube with the right-sided pneumothorax interval improvement.

On the 21st day, he developed a small right-sided pneumothorax and bilateral subcutaneous emphysema involving the neck and chest wall (Figure 5). Ventilator settings at that time were pressure-regulated volume control mode with a RR of 32 breaths/min, PEEP 12 cm H_2_O, TV 340 mL, FiO_2_ 100%, and IT 0.7 s. The highest PEEP on the ventilator since intubation was 12 cm H_2_O. Tube thoracostomy was performed with a 20 Fr surgical catheter (Figure 6). He underwent tracheostomy placement eventually.

He had a prolonged ICU stay of 58 days. His hospital stay was further complicated by extended-spectrum beta-lactamase (ESBL) positive *Escherichia coli* in the urine and sputum, *Pluralibacter gergoviae* in the sputum, and pleural effusion with Candida in the pleural fluid. He was treated with high-dose steroids, hydroxychloroquine, tocilizumab, convalescent plasma, broad-spectrum antibiotics, and antifungals. Due to worsening clinical status and poor prognosis, the family opted for comfort care.

Case 3

A 41-year-old Caucasian male with no significant medical history presented to the ED with cough and worsening shortness of breath for one week after he tested positive for COVID-19 at an outpatient facility (Table 1). He was hypoxic to 79% on arrival following which 6 L of oxygen via nasal cannula and subsequently high flow nasal cannula (HFNC) was initiated to maintain an oxygen saturation of more than 90%. Initial CXR showed bilateral multifocal pulmonary infiltrates. He was admitted to telemetry for the management of acute hypoxic respiratory failure due to COVID-19 pneumonia.

**Figure 7:**
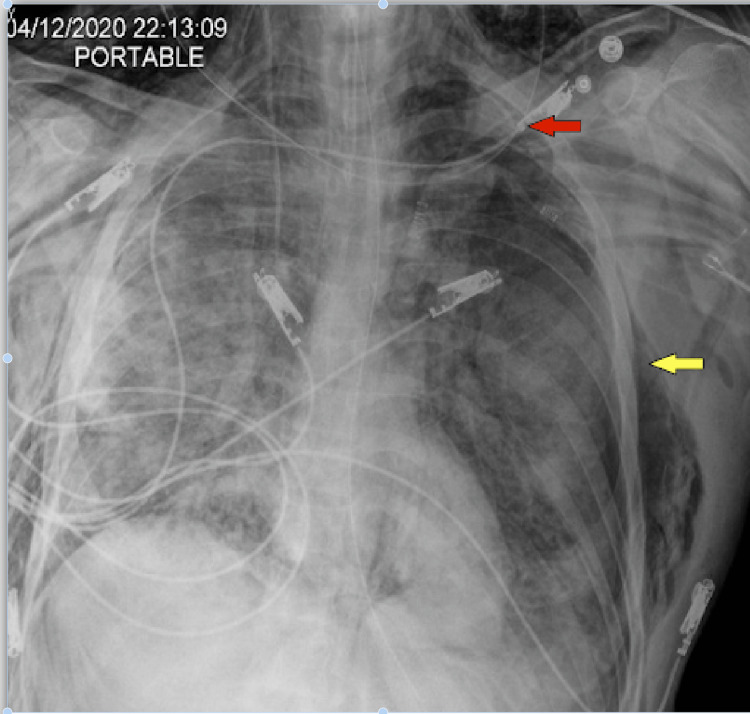
​​Moderate left-sided pneumothorax (red arrow), pneumomediastinum, and bilateral subcutaneous emphysema (yellow arrow).

**Figure 8:**
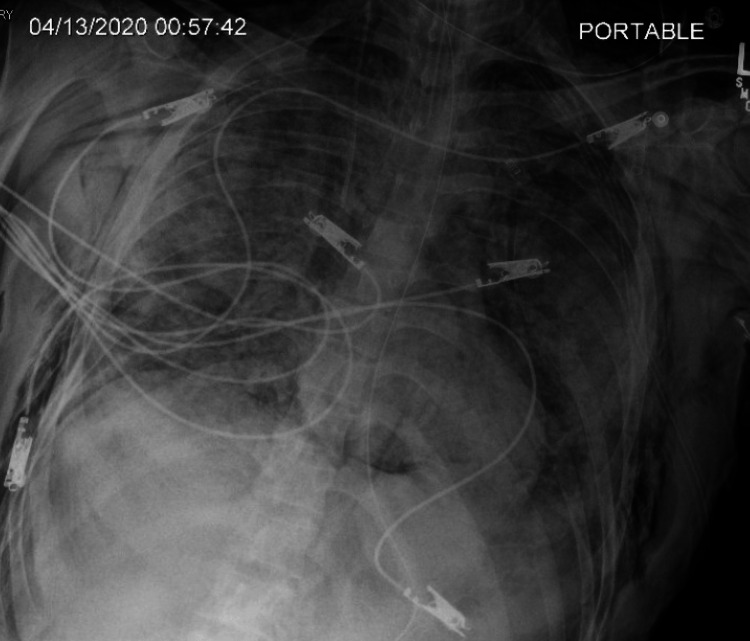
New left-sided chest tube with a left apical pneumothorax, slightly decreased from previous, now considered to be small.

**Figure 9:**
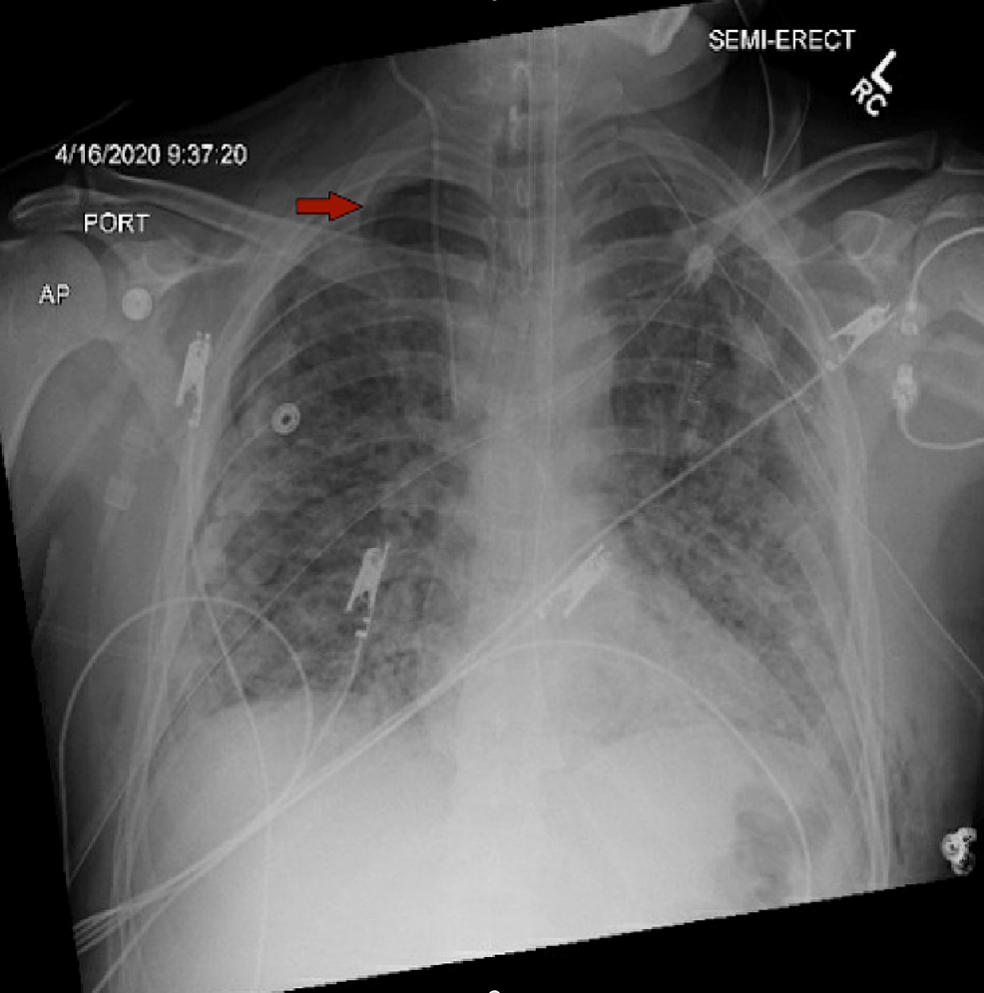
New right-sided small pneumothorax (arrow).

**Figure 10:**
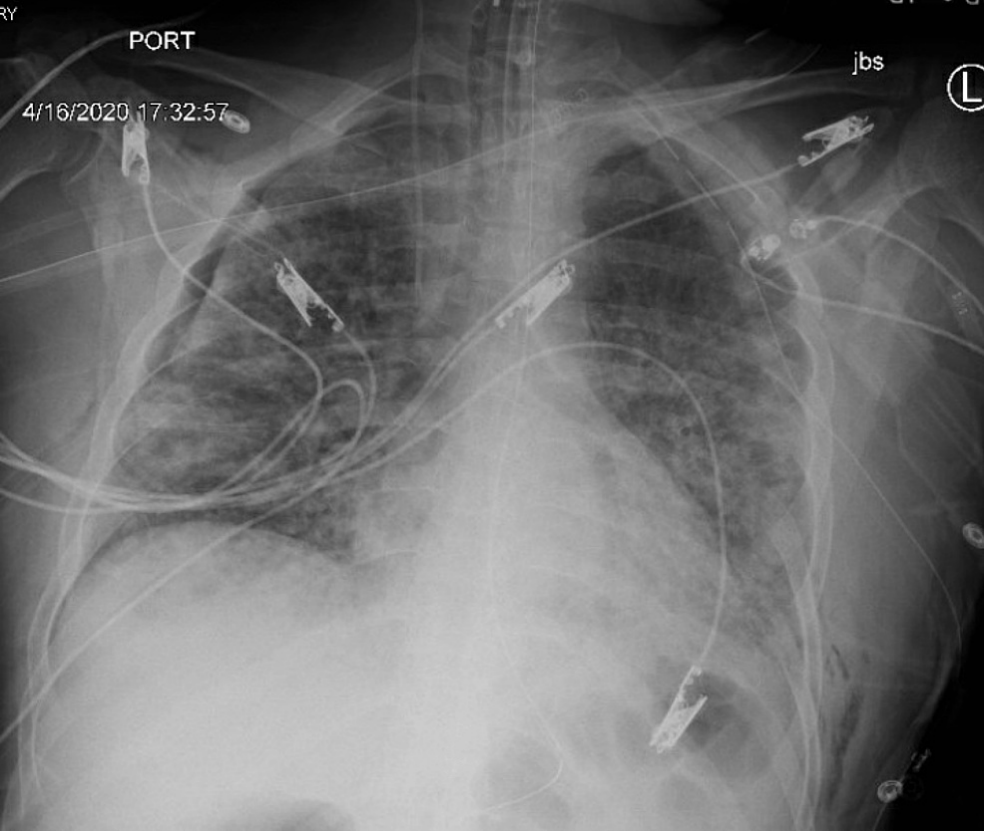
New right-sided chest tube with unchanged small right apical pneumothorax.

**Figure 11:**
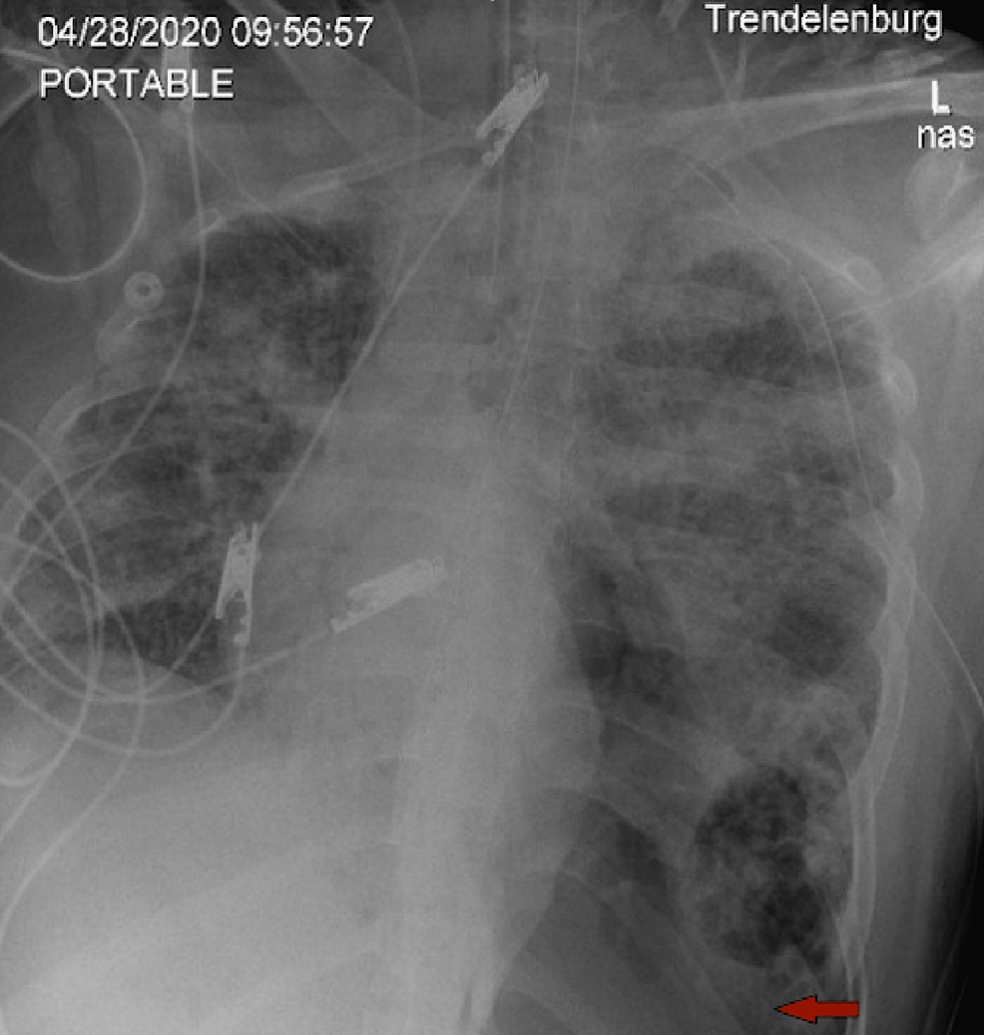
Large air-containing loculated pneumothorax (arrow) in the lower half of left hemothorax.

**Figure 12:**
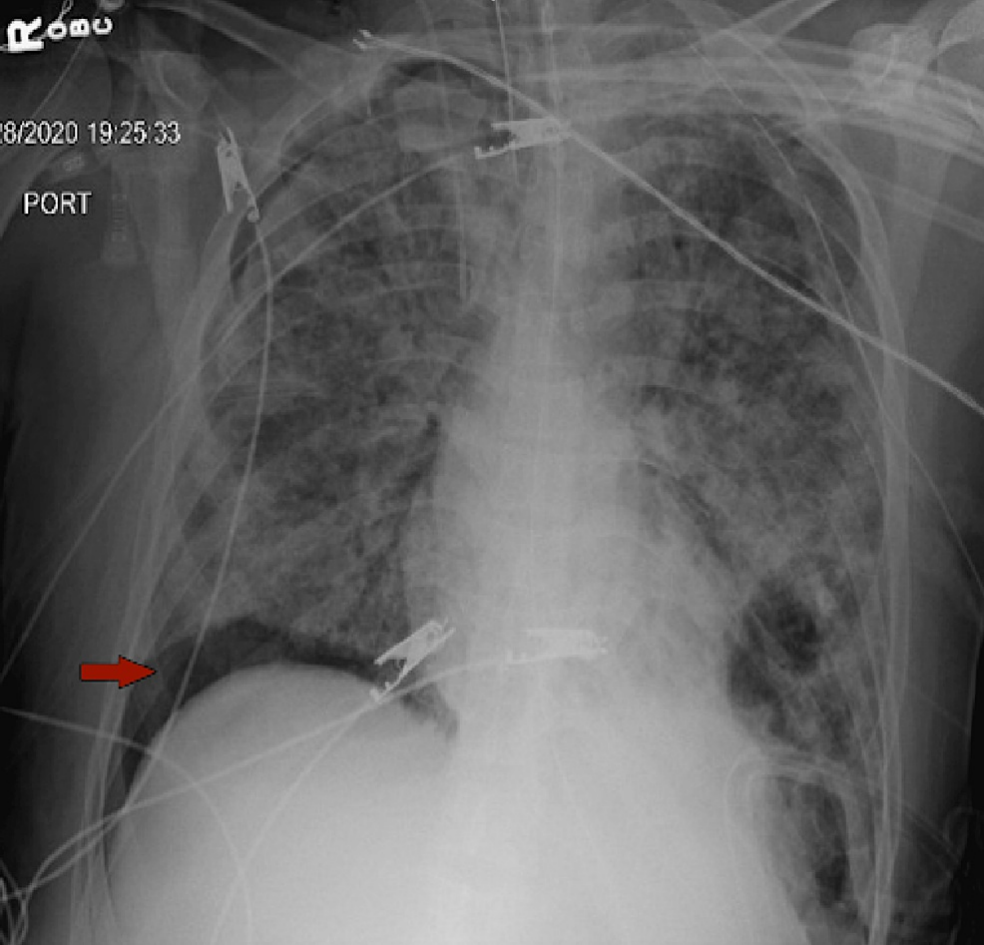
Right-sided pneumothorax (arrow) which appears more prominent.

On day six, he was intubated to volume control settings due to worsening hypoxemia on HFNC and was transferred to ICU. Three days after intubation, a CXR was obtained due to worsening hypoxia on mechanical ventilation which showed left pneumothorax and bilateral subcutaneous emphysema (Figure 7), and a 24 Fr chest tube was placed on the left side (Figure 8). Ventilator settings at that time were pressure control/assist control (PC/AC) mode, RR of 32 breaths/min, peak inspiratory pressure (PIP) 30 mm H_2_O, IT 0.9 s, PEEP 8 cm H_2_O, and the FiO_2_ 90% (Table 2). Three days later, he developed another pneumothorax on the right side (Figure 9) and a tube thoracostomy was performed with a 28 Fr surgical catheter (Figure 10). In due course, he developed another bilateral pneumothorax and another left and right chest tube were placed, making it a total of four chest tubes, two on each side (Figures 11, 12).

His ICU course was further complicated by *Escherichia coli* in sputum and bilateral peroneal and soleal vein thrombosis. He was treated with high-dose steroids, hydroxychloroquine, tocilizumab and convalescent plasma, heparin infusion, nitric oxide, and a high-frequency oscillator. After 21 days of being on a ventilator, he underwent cardiac arrest and unfortunately passed away despite cardiopulmonary resuscitation.

Case 4

A 48-year-old Hispanic male with a medical history of hypertension, diabetes mellitus, hyperlipidemia, end-stage renal disease on dialysis, and peripheral vascular disease presented to the ED with fever, cough, and shortness of breath for 11 days (Table 1). On admission, he was found to be hypoxic to 70% on room air and was transitioned to HFNC. Laboratory investigations were significant for lymphopenia of 0.32×10^3^/mm^3^, D-dimer of 1484 ng/mL, CRP 300 mg/L, LDH 756 U/L, and ferritin 7500 ng/mL (Table 2). CXR showed diffuse bilateral opacities representing multifocal pneumonia. COVID PCR was positive. While he was in ED, he had a cardiac arrest, requiring intubation to volume control settings, and was shifted to the ICU.

**Figure 13:**
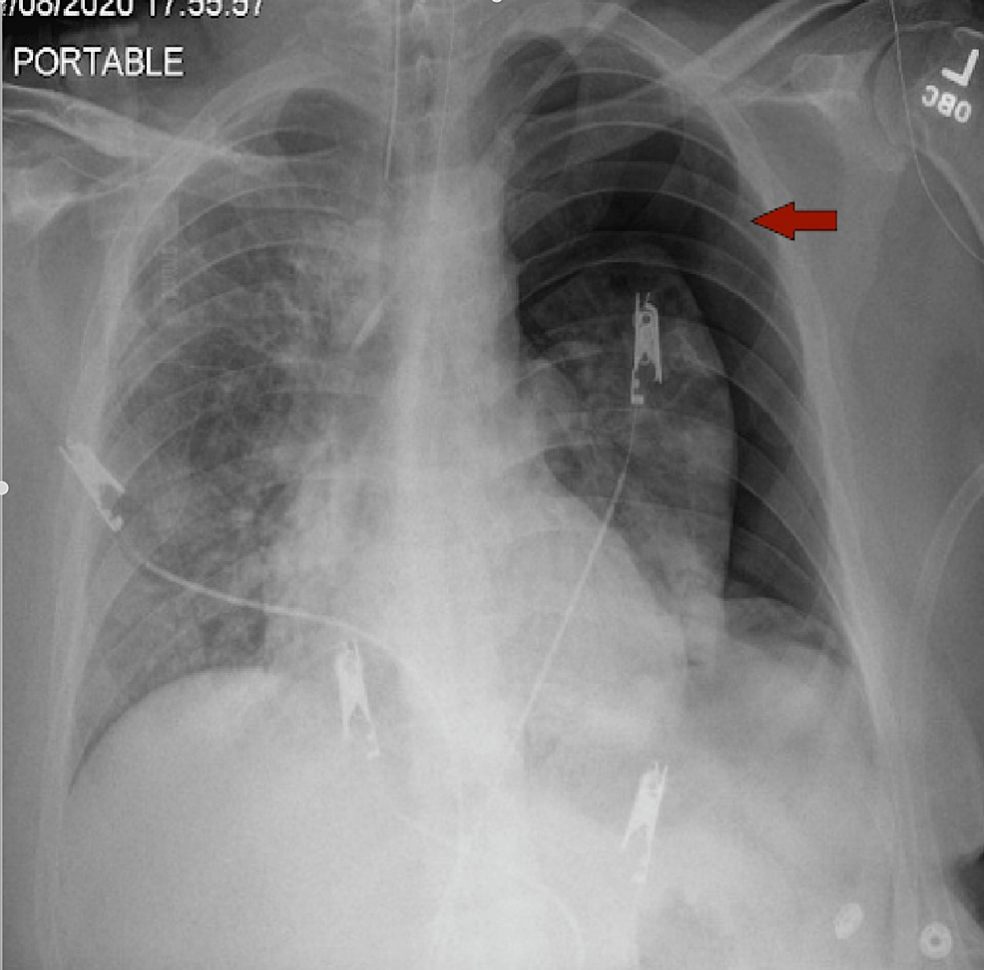
New large left pneumothorax (arrow), possibly tension pneumothorax given the mediastinal shift.

**Figure 14:**
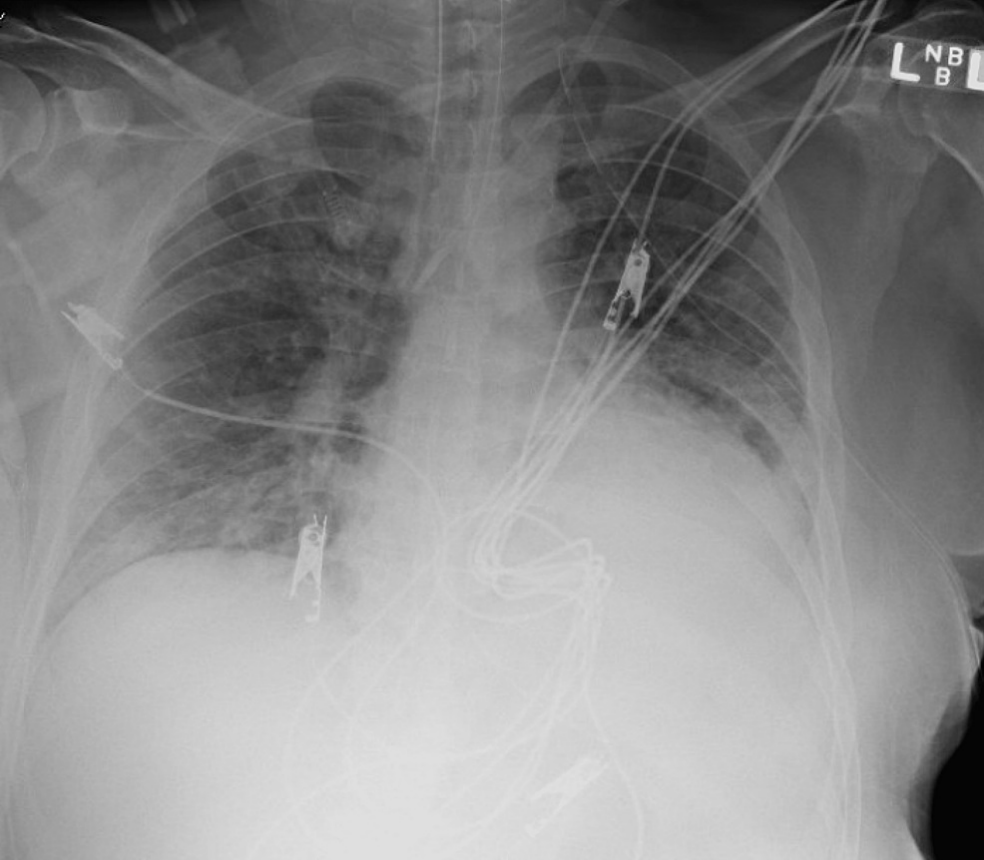
New left chest tube with the tip at the left lung apex.

On day two, due to hypotension and persistent hypoxia on the ventilator, CXR was done which revealed a new large left pneumothorax (Figure 13). Ventilator settings at that time were volume control mode, with a RR of 20 breaths/min, PEEP 10 cm H_2_O, TV 500 mL, FiO_2_ 100%, and IT 1 s. The highest PEEP on the ventilator since intubation was 10 cm H_2_O. Tube thoracostomy was performed with a 20 Fr surgical catheter (Figure 14). He was treated with hydroxychloroquine. On day seven, he underwent a cardiac arrest and passed away despite resuscitative efforts.

Case 5

A 48-year-old Hispanic male with a medical history of hyperlipidemia presented with 10 days of progressive dyspnea. On arrival, he was hypoxic to 71% on room air (Table 1). Laboratory results showed leukocytosis of 13.3×10^3^/mm^3 ^and lactic acidosis of 4.3 mmol/L. Inflammatory markers were elevated with a D-dimer of 6534 ng/ mL, CRP 282 mg/L, LDH 866 U/L, and ferritin 455 ng/mL (Table 2). CXR showed patchy bilateral pulmonary airspace opacities compatible with multifocal atypical pneumonia. COVID-19 PCR was positive. He was initially managed with HFNC, but due to worsening oxygen saturation, he was intubated, placed on mechanical ventilation with volume control mode, and admitted to ICU for further care.

**Figure 15:**
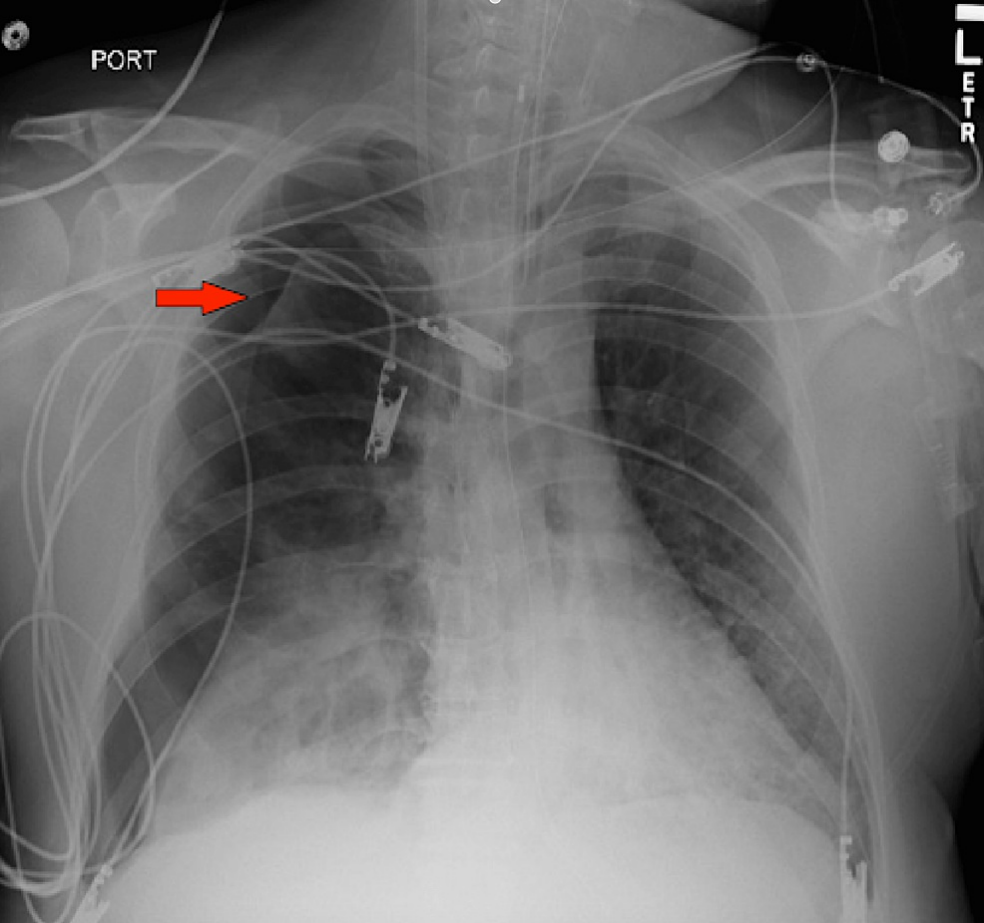
New moderate-sized right pneumothorax with a new mild leftward mediastinal shift concerning tension pneumothorax (arrow).

**Figure 16:**
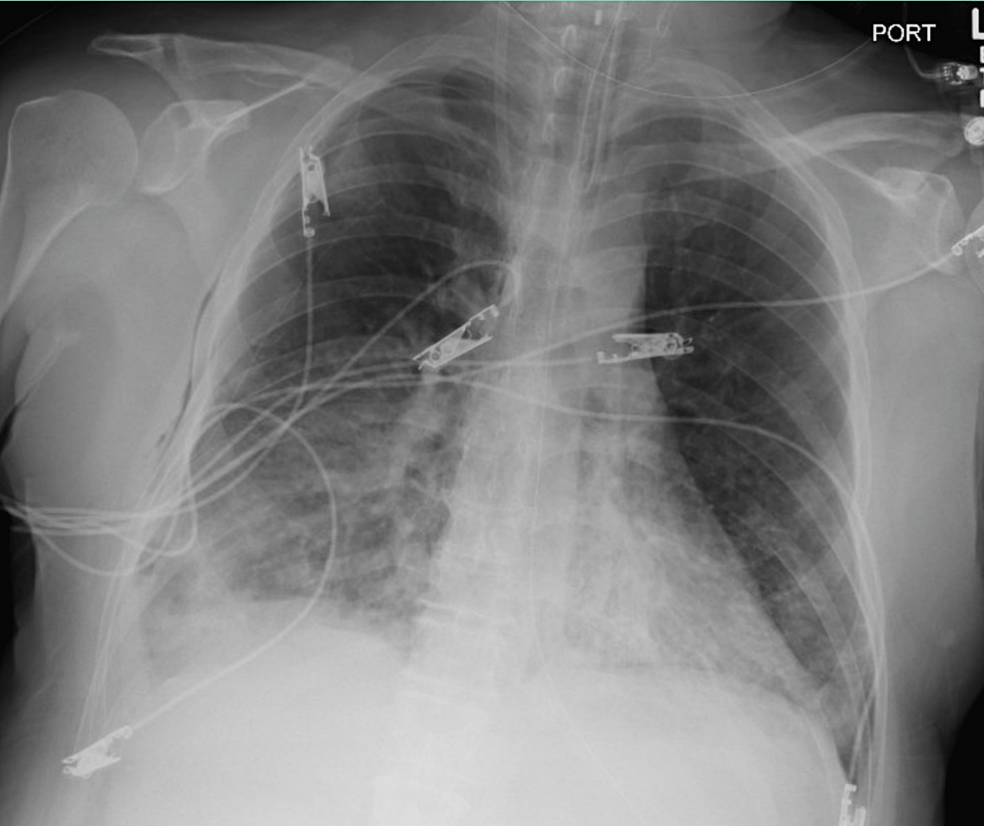
Interval placement of right chest tube with the tip in the right upper lung field. Minimal right hemithorax subcutaneous emphysema.

On day eight of mechanical ventilation, his oxygen saturation started to drop on the ventilator. CXR revealed a new moderate-sized right pneumothorax with a new mild leftward mediastinal shift concerning tension pneumothorax (Figure 15). Ventilation settings around that time were volume control mode, RR of 30 breaths/min, PEEP 8 cm H_2_O, TV 350 mL, FiO_2_ 100%, PIP 28 mm H_2_O, and IT 0.9 s. Tube thoracostomy was performed with a 12 French (Fr) surgical catheter (Figure 16).

He had a prolonged ICU course complicated by superimposed *Klebsiella pneumoniae* and *Serratia marcescens* pneumonia and *Klebsiella pneumoniae* bacteremia. He was treated with norepinephrine, vasopressin, steroids, hydroxychloroquine, tocilizumab, broad-spectrum antibiotics, convalescent plasma, continuous venovenous hemodialysis (CVVHD), and heparin infusion for acute lower extremity deep venous thrombosis in bilateral gastrocnemius and peroneal veins. He was on mechanical ventilation for a total of 19 days. On day 20 of hospitalization, he had a cardiac arrest and eventually passed away.

Case 6

A 70-year-old Caucasian male with a medical history of hypertension, type 2 diabetes mellitus, hyperlipidemia, and renal cell carcinoma status post unilateral nephrectomy presented to the emergency department with fever, dyspnea, and weakness for one week (Table 1). On presentation, his oxygen saturation was 63% on room air. Laboratory studies showed lactic acidosis of 3 mmol/L. Inflammatory markers were elevated with a D-dimer of 299 ng/mL, CRP 198 mg/L, and LDH 459 U/L (Table 2). COVID-19 PCR was positive. CXR showed mild right and moderate left lung airspace opacities suspicious for atypical multifocal pneumonia. Initially, he was placed on an HFNC but his respiratory status deteriorated the next day. He was intubated for worsening hypoxia and placed on mechanical ventilation with volume control mode.

**Figure 17:**
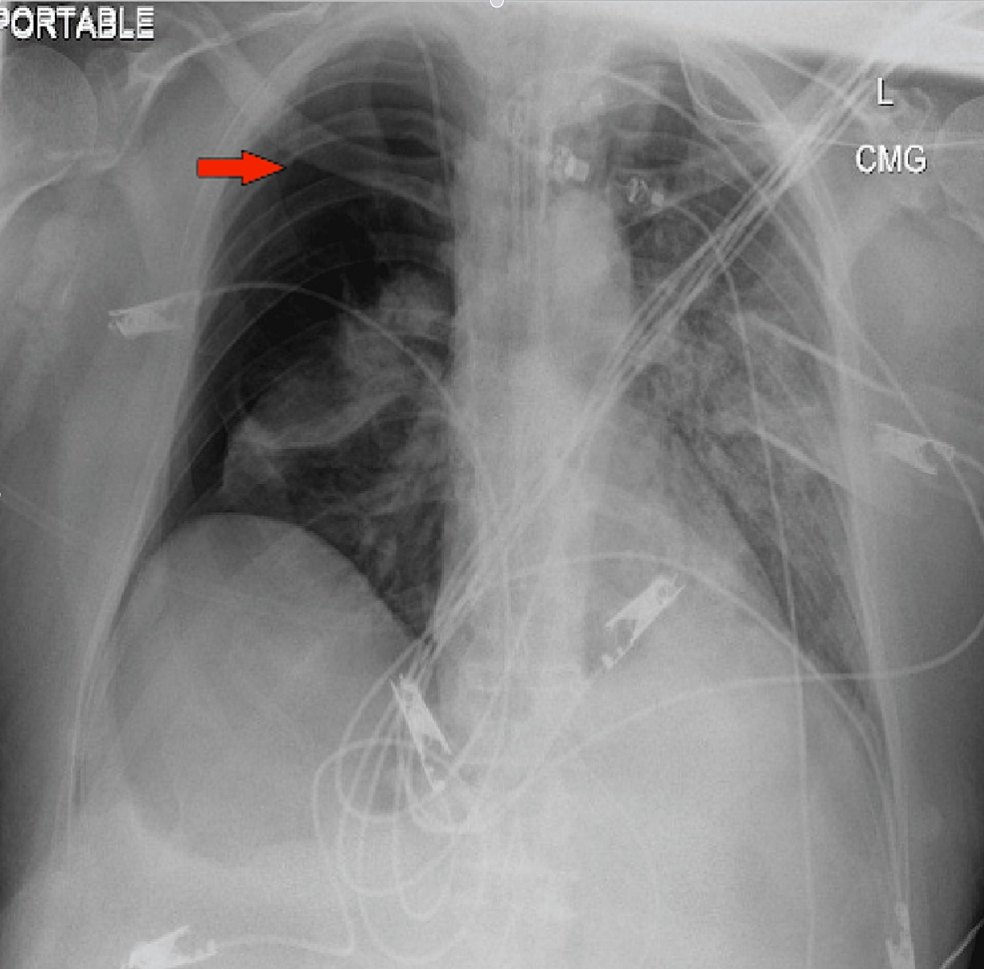
​​Moderate right pneumothorax (arrow).

**Figure 18:**
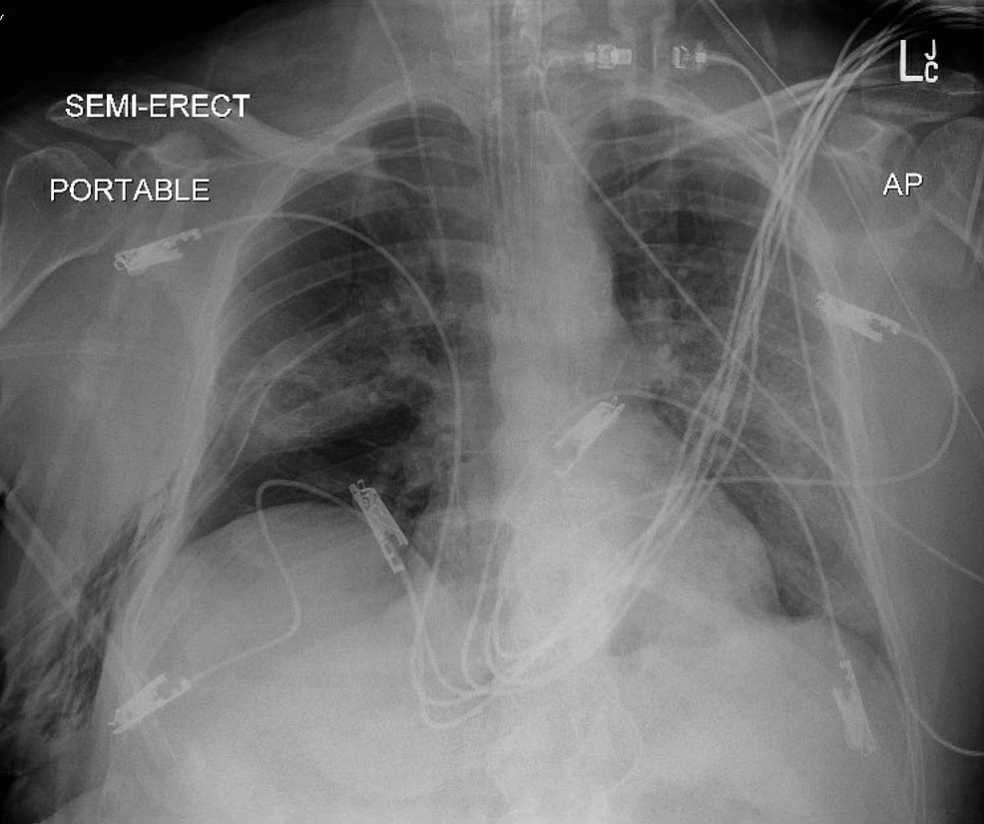
Interval placement of right chest tube with decreased, but not resolved, right pneumothorax.

On day two of mechanical ventilation, the patient became hypoxic on ventilator. CXR showed moderate right pneumothorax (Figure 17). Ventilation settings around that time were volume control mode, RR of 28 breaths/min, PEEP 14 cm H_2_O, TV 350 mL, FiO_2_ 90%, and IT 0.8 s. The highest PEEP on the ventilator since intubation was 16 cm H_2_O. Tube thoracostomy was performed with a 24 French (Fr) surgical catheter (Figure 18).

The hospital course was complicated by coagulase-negative Staphylococcus bacteremia. His treatment regimen included vasopressors, steroids, broad-spectrum antibiotics, hydroxychloroquine, convalescent plasma, and CVVHD. He was intubated for a total of five days. On day six of hospitalization, he had a cardiac arrest and eventually passed away.

Case 7

A 61-year-old Caucasian female with a medical history of chronic obstructive pulmonary disease (COPD) asthma overlap, a former smoker with 16 pack-year smoking history, hypertension, and type 2 diabetes mellitus presented to the ED with nausea, vomiting, loss of appetite, and lightheadedness for eight days (Table 1). Vitals on presentation were significant for hypoxia with an oxygen saturation of 65% on room air. Inflammatory markers were elevated with a D-dimer of 813 ng/mL, CRP 254 mg/L, and LDH 534 U/L (Table 2). CXR showed diffuse bilateral patchy airspace opacities. COVID-19 PCR was positive. She was initially placed on an HFNC. On day three of hospitalization, she became hypoxic on HFNC requiring intubation and mechanical ventilation with volume control settings. She was then shifted to the ICU.

**Figure 19:**
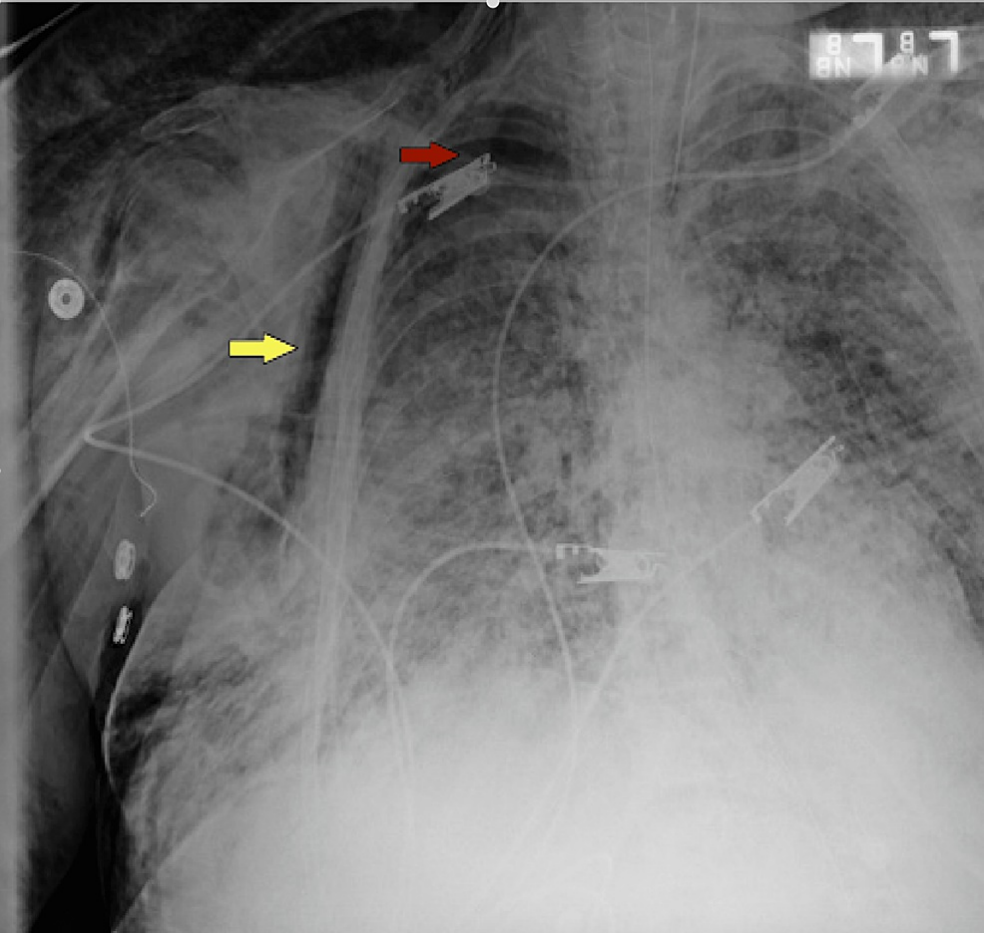
Increased extensive bilateral lung airspace opacities. New small right (red arrow) apical lucency with suspected small right apical pneumothorax (5-10%). New extensive bilateral chest/right upper extremity subcutaneous emphysema (right greater than left) (yellow arrow).

**Figure 20:**
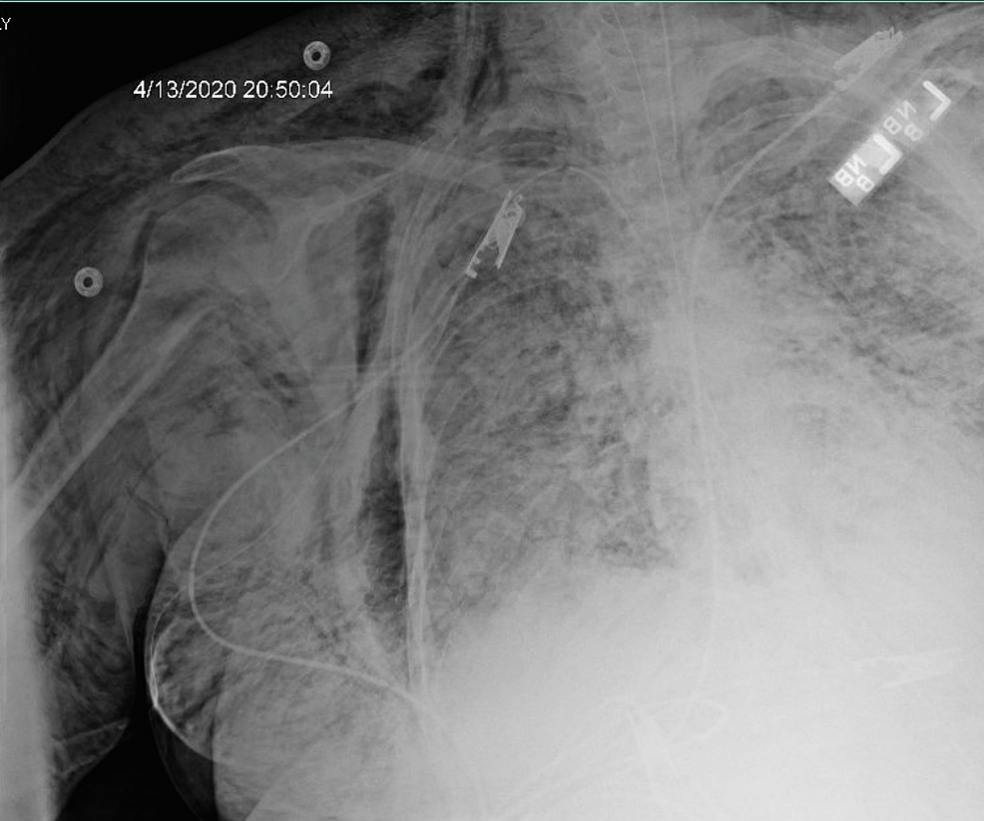
Interval placement of right-sided chest tube without definite residual right apical pneumothorax.

On day five of mechanical ventilation, she became hypoxic on the ventilator. CXR showed new small right apical lucency with suspected small right apical pneumothorax and new extensive bilateral chest/right upper extremity subcutaneous emphysema, right greater than left (Figure 19). Ventilator settings around that time were volume control mode, RR of 30 breaths/min, PEEP 13 cm H_2_O, TV 450 mL, FiO_2_ 50%, and IT 1 s. The highest PEEP on the ventilator since intubation was 15 cm H_2_O. Tube thoracostomy was performed with a 12 French (Fr) surgical catheter (Figure 20).

She was on norepinephrine, vasopressin, steroids, broad-spectrum antibiotics, and hydroxychloroquine. Due to an overall poor prognosis, on day 11 of hospitalization, the patient’s care was transitioned to comfort care measures only.

Case 8

A 66-year-old Hispanic male with a medical history of hypertension presented with shortness of breath and a non-productive cough for a month (Table 1). He was hypoxic to 75% on room air. Laboratory results showed leukocytosis of 11.7×10^3^/mm^3^ and lactic acidosis of 3.6 mmol/L. Inflammatory markers were elevated with a D-dimer of 516 ng/mL, CRP 72 mg/L, and LDH 521 U/L (Table 2). CXR on admission was consistent with COVID-19 pneumonia showing multifocal patchy densities.

**Figure 21:**
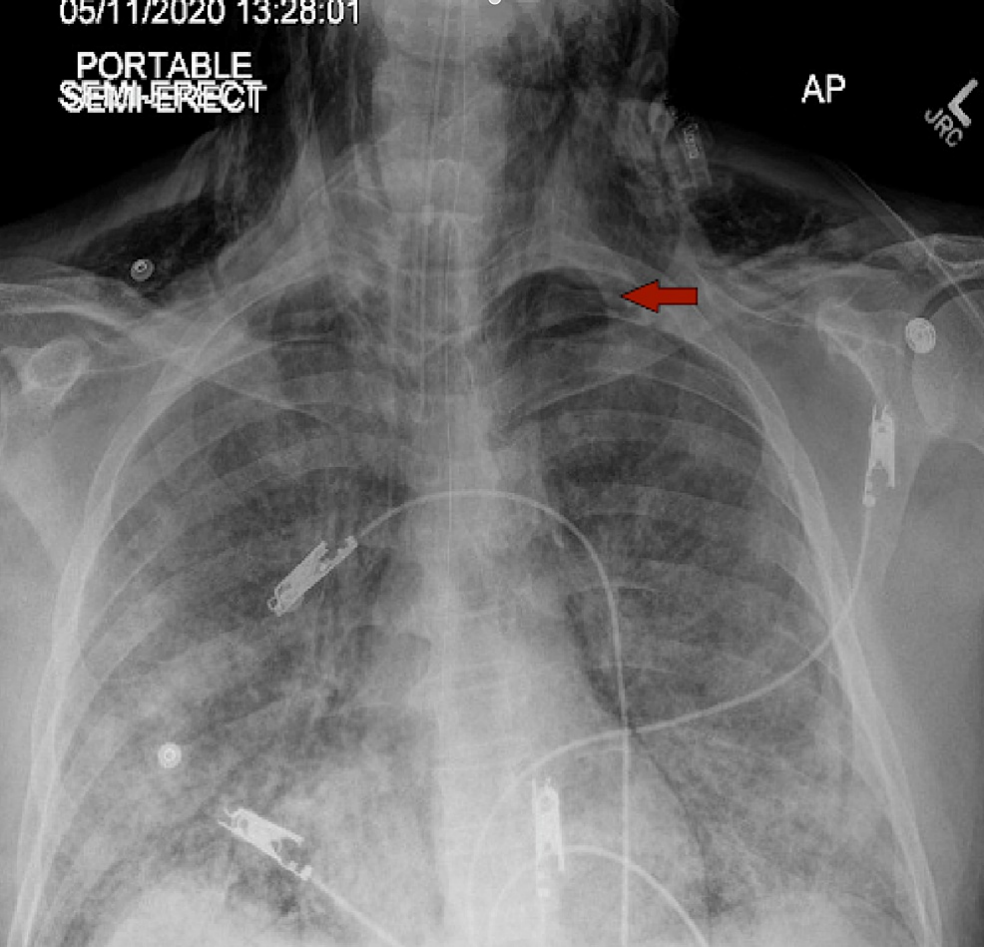
New small left-sided pneumothorax (arrow). New pneumomediastinum and subcutaneous emphysema.

**Figure 22:**
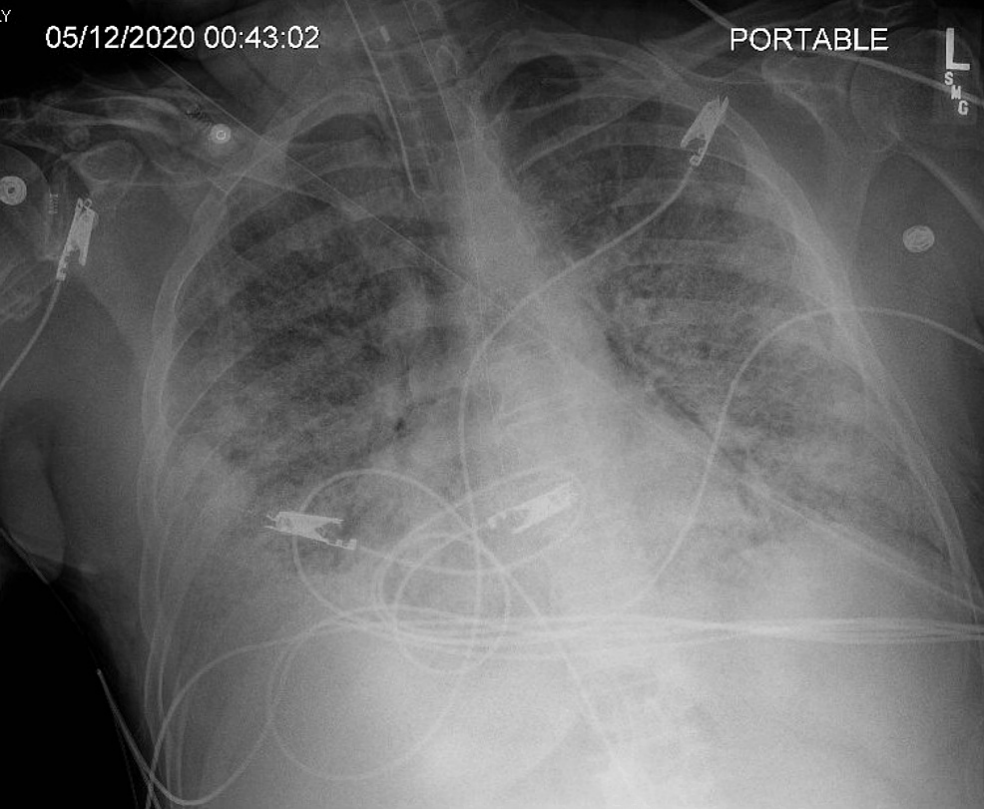
Trace to small left pneumothorax decreased in size. Subcutaneous emphysema at the lower neck has decreased. There is a left-sided chest tube.

He was initially placed on HFNC and admitted for management of acute hypoxic respiratory failure due to COVID-19 pneumonia. On day 14 of hospitalization, he became hypoxic, required intubation, and was placed on pressure-regulated volume control mode on the ventilator. The next day, CXR showed a new small left-sided pneumothorax, new pneumomediastinum, and subcutaneous emphysema (Figure 21), requiring tube thoracostomy with an 18 French (Fr) surgical catheter (Figure 22). Ventilator settings around that time were pressure-regulated volume control mode, RR of 30 breaths/min, PEEP 12 cm H_2_O, TV 400 mL, FiO_2_ 100%, and IT 1.15 s. The highest PEEP on the ventilator since intubation was 15 cm H_2_O.

His hospital course was prolonged and complicated by upper gastrointestinal bleeding. He was treated with broad-spectrum antibiotics, hydroxychloroquine, steroids, vasopressors, convalescent plasma, and CVVHD because of acute renal failure due to acute tubular necrosis. He was on the ventilator for four days. On day 18 of hospitalization, he had a cardiac arrest and passed away.

Case 9

A 58-year-old Hispanic male with no significant medical history presented with dyspnea for a two-week duration (Table 1). On arrival, he was hypoxic to 83% on room air and was placed on HFNC for acute hypoxic respiratory failure. Blood work was remarkable for lymphopenia with an absolute lymphocyte count of 0.55×10^3^/mm^3^ and lactic acidosis of 2.8 mmol/L. Inflammatory markers were elevated with a CRP 288 mg/L and LDH 612 U/L (Table 2). CXR showed diffuse bilateral multifocal airspace opacities.

**Figure 23:**
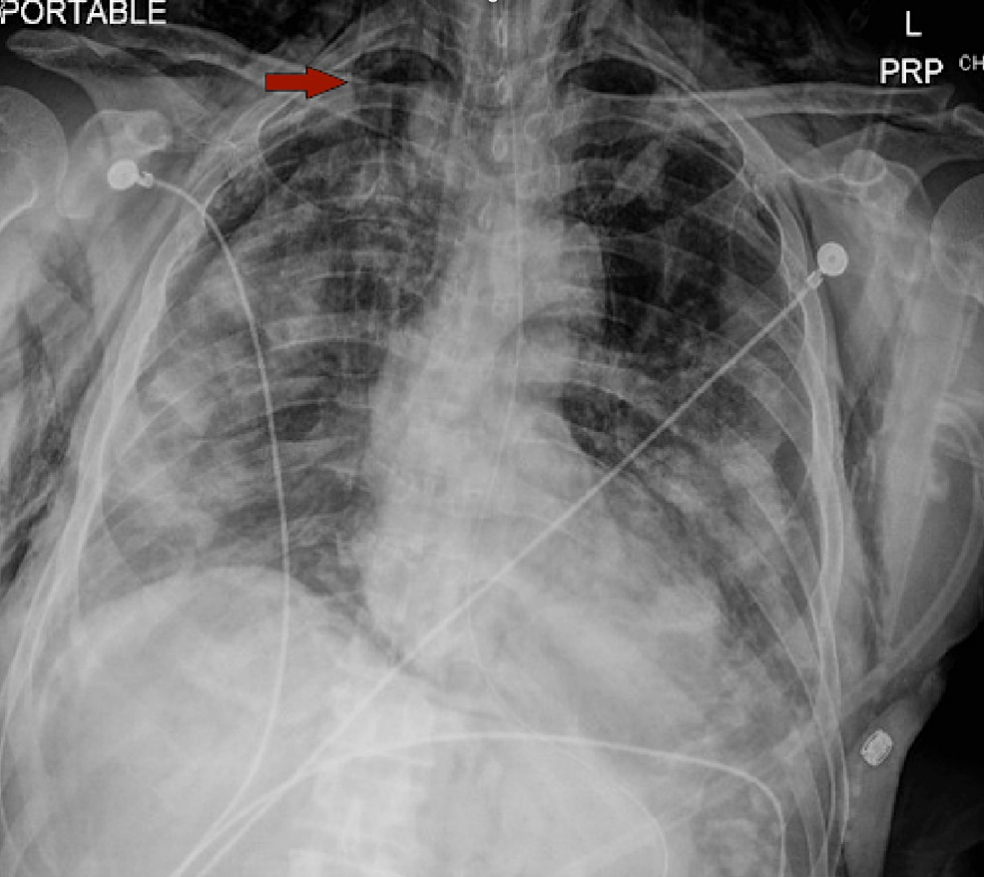
New small right apical-lateral pneumothorax (arrow). New pneumomediastinum. New extensive bilateral neck/chest subcutaneous emphysema.

On day three of admission, he was intubated to pressure control due to worsening hypoxia. After six days of being on a ventilator, he became hypoxic, tachycardic, and hypotensive. Tense swelling over the chest and neck was noticed. CXR done at that time showed a new small right apical-lateral pneumothorax, pneumomediastinum, and extensive bilateral neck/chest subcutaneous emphysema (Figure 23). Ventilator settings at that time were PC/AC, RR of 28 breaths/min, PIP 32 mm H_2_O, IT 1 s, PEEP 8 cm H_2_O, and FiO_2_ 45%. Tube thoracostomy was done which improved the vitals.

He had a prolonged ICU stay of 42 days. He received high-dose steroids, hydroxychloroquine, tocilizumab, remdesivir, convalescent plasma, and heparin infusion for bilateral soleal veins thrombosis. On day 44 of hospitalization, he suffered a cardiac arrest and passed away.

## Discussion

Patients with COVID-19 appear to be at an increased risk of pneumothorax due to severe pulmonary inflammation. It may be seen in any stage of the disease and may or may not be associated with the severity of the illness. As in our case series, it was not correlated with previous lung disease, smoking status, comorbidities, or days on the ventilator. All of our patients however suffered from severe ARDS due to COVID-19 pneumonia and hence severe lung inflammation, setting up an optimal environment for the pathogenesis of pneumothorax. There have been many other case reports/series, retrospective cohort studies, and meta-analyses that have highlighted this phenomenon of pneumothorax and pneumomediastinum in COVID-19 patients [1-12]. It is said that 1% of all patients develop pneumothorax with COVID-19, although almost 7.5% of patients who are admitted develop pneumothorax with a mortality rate of about 58%. However, approximately 10% of critically ill patients in the ICU and up to 15% of those who are mechanically ventilated experienced pneumothorax with mortality from 66% to 80% in some studies, which significantly increased compared to those who do not develop pneumothorax and increase the length of stay as well [6,12,13].

It is likely that the pathogenesis of pneumothorax in COVID-19 is multifactorial. It is thought that there are four key factors in the pathogenesis of pneumothorax. These include the following: increased alveolar pressure, most probably due to recruitment positioning or plateau pressure (Pplat) of 35 cm H_2_O or greater; increased negative pleural pressure due to respiratory rates of 30 per minute or greater, severe cough, forced inhalation or paradoxical thoracoabdominal motion; alveolar shear stress seen as heterogeneous attenuation or patchy infiltrates on chest imaging; and finally, changes in lung structure and function due to various disease processes seen as consolidation, fibrosis, cysts, or emphysema on radiographic imaging [12]. Based on the pathogenesis of COVID-19, it is of no surprise that lung structure and function are compromised as seen most often on chest imaging as bilateral peripheral ground-glass opacities with or without consolidation transforming into reticular opacities associated with fibrosis, volume loss, architectural distortion, and traction bronchiectasis over weeks time [14].

According to the National Institute of Health (NIH); National Heart, Lung, and Blood Institute (NHLBI) ARDS clinical network mechanical ventilation protocol, the gold standard is to treat with PEEP in order to increase oxygenation and decrease mortality. Two following strategies may be utilized: a high PEEP with low FiO_2_, or a low PEEP with high FiO_2_. However, the literature suggests that there is no significant difference between the outcomes of the two strategies [15]. Regardless of the PEEP used in the ventilation of COVID-19 patients, it predisposes the already diseased lungs to ventilator-associated lung injury (VALI) in the form of macro or microbarotrauma. Utilizing lung protective measures such as limiting TV to 4-8 mL/kg ideal body weight, Pplat below 30 cm H_2_O, and driving pressure (Pplat - PEEP) between 13 and 15 cm H_2_O has been shown to decrease VALI and overall mortality [16-19]. However, a retrospective study found that despite maintaining acceptable ventilator settings, 40% of patients experienced barotrauma and there are patients who even experience pneumothorax on room air [3,16-19]. This further strengthens the notion that pneumothorax is multifactorial in COVID-19. A few less well-known mechanisms have been proposed, including ischemic injury due to pulmonary microthrombi predisposing to diffuse alveolar damage and rupture; as well as type II pneumocyte damage causing pulmonary interstitial emphysema due to COVID-19 viral entry via angiotensin converting enzyme-2 (ACE-2) receptors [16,20-22]. In the mentioned retrospective study, barotrauma was most common in those with diabetes mellitus and hypertension as ACE-2 receptors are upregulated in these conditions, supporting that theory [16].

In our case series of patients with COVID-19 who develop a pneumothorax, 67% had hypertension and 55% had diabetes mellitus, supporting the above-mentioned pathophysiology leading to pneumothorax. All of our patients except for one had a history of smoking and chronic lung disease, highlighting that previous lung structure and function are less likely a predisposing factor. In our case series, patients were relatively on higher PEEP’s in the range of 10-16 cm H_2_O. The median number of days on the ventilator was 19 days (four to 58 days) and the median time from ventilator to pneumothorax was seven days (one to 21 days). The mortality was noted to be 100%. This is similar to another study where length of stay (LOS) was 25 days and time to pneumothorax was 5.3 days, although mortality was 53% [16,20].

COVID-19 is a novel illness with no guidelines specific to the management of the disease with respect to mechanical ventilation parameters. Therefore, intensivists should be cautious while maintaining PEEP in such patients and all efforts should be made to prevent pneumothorax in COVID patients. However, patients may progress rapidly into worsening respiratory failure due to ARDS, which can make it challenging to identify pneumothorax. Hence, early imaging with CXR or point-of-care ultrasound can assist in making a prompt diagnosis leading to earlier management and hopefully a better outcome for the patient [16]. In terms of management, one study found that large-bore thoracostomy tubes had fewer complications than small-bore chest tubes [13]. However, there may be a higher risk of aerosolization of COVID-19 particles during the surgical placement of a thoracostomy tube and therefore the Seldinger’s technique using a guide-wire can be performed in lesser time and may help reduce exposure to the aerosolized virus [16].

## Conclusions

Due to the significant lung damage caused by COVID-19 pneumonia and the high ventilator settings needed to treat it, pneumothorax can be a potentially fatal consequence in these patients. Our case series adds to the medical literature on barotrauma and in particular pneumothorax in COVID-19. We suspect that COVID-19 may predispose patients to barotrauma in ways that differ from other pneumonia-induced ARDS and may have separate pathophysiology from VALI. However, we are in need of larger studies to add to our knowledge of the predisposing factors, incidence, management, and outcomes of these patients. At this time, we should be prepared and ready to handle this fatal outcome in our COVID-19 patients.
